# Detection and diagnosis of cleidocranial dysplasia by panoramic radiography: a retrospective study

**DOI:** 10.1186/s12903-022-02610-7

**Published:** 2022-12-01

**Authors:** Yuchao Shi, Zelin Ye, Yuanyuan Liu, Hu Wang, Meng You

**Affiliations:** State Key Laboratory of Oral Diseases and National Clinical Research Center for Oral Diseases and Department of Oral Radiology, West China Hospital of Stomatology, Sichuan University, No. 14, Section 3, Ren Min South Road, Chengdu, 610041 Sichuan China

**Keywords:** Cleidocranial dysplasia, Zygomatic arch, Mandible, Panoramic radiography

## Abstract

**Background:**

Cleidocranial dysplasia (CCD) is a rare and underdiagnosed congenital disorder in dentistry. The purpose of this study was to illustrate and quantify the maxillofacial bone abnormalities detected on panoramic radiographs from a relatively large retrospective case series and to provide a series of diagnostic references for dentists to indicate the presence of disease and help in making an early and accurate diagnosis.

**Methods:**

The dental panoramic radiographs of thirty CCD patients aged 11 to 45 years (18 males and 12 females) were examined retrospectively. The dentition states, including supernumerary teeth and impacted teeth, were recorded. Twelve quantified measurements were adopted to determine the abnormalities of maxillofacial bones, including the degree of the zygomatic arch downward bend, bicondylar breadth, ramal height, mandibular height, mandibular aspect ratio, mandibular body height, condylar height, coronoid height, distance between the coronoid process and the condyle, bigonial width, gonial angle and best-fit gonial circle diameter. The Wilcoxon rank-sum test was used to compare the findings of the CCD patients with those of their matched controls (n = 300).

**Results:**

Supernumerary teeth were detected in 27 patients (90.0%), and all 30 patients presented impacted teeth. Compared to the matched controls, the CCD patients had a significantly larger degree of zygomatic arch downward bend (ZAD), a larger diameter of the best-fit gonial circle (BGC), and a shorter distance between the coronoid process and the condyle (DCC) in panoramic radiographs (P < 0.001). According to the reference cutoff values established from the 5th or 95th percentile of the measurements in the control group, ZAD higher than 6.90 mm, DDC less than 22.37 mm and BGC higher than 52.41 mm were significantly associated with the CCD features identified. Other panoramic measurements were not significantly different between the two groups.

**Conclusions:**

Panoramic radiographs had great value in the diagnosis of CCD. In this study, we identified some dental and maxillofacial features on panoramic radiographs from a relatively large retrospective case series of CCD. A series of reliable quantitative indicators were provided for dentists that can indicate the presence of disease and improve the diagnostic specificity.

## Background

Cleidocranial dysplasia (CCD) is a rare and underdiagnosed congenital disorder that primarily affects the development of bones and teeth. Currently, the disease still lacks a pathognomonic “gold standard” due to its phenotypic variability. The clinical diagnosis can be made based on a set of physical and radiological characteristics, which include underdeveloped or absent clavicles, delayed closure of fontanelles, and dental and maxillofacial abnormalities [1, 2]. As the most well-known genetic predisposing factor for this disease, mutational analysis of the Runt-related transcription factor 2 (RUNX2) gene may be utilized for diagnostic confirmation [3–5]. However, not all RUNX2 mutations are identified on standard DNA sequencing. In patients with a clinical diagnosis of CCD, the RUNX2 mutations are detected in about 60–70%. The genetic conditions in the remaining 30–40% of the cases are still unknown [1, 6, 7]. For dentists the diagnosis can be more difficult because the limited availability of diagnostic modality in most of the dental institutions.

Patients with CCD commonly present with symptoms of deciduous tooth retention, delayed eruption of permanent dentition, and supernumerary teeth. Hence, dentists are often the first health professionals to encounter patients with the potential diagnosis. In most cases, panoramic radiographs are often used as the initial evaluation image, which has the particular advantages of a low radiation dose and broad coverage of the jaws [8]. In addition to the evaluation of the dentition, panoramic radiography can also provide the visualization of adjacent structures, such as the mandible, maxilla, zygomatic bone and temporo-mandibular joints. For dentists, whether panoramic radiography can provide a responsible assessment of CCD is a key question.

Although several studies have described panoramic features of CCD, most of them based on only a few cases, and no study has had standardized measurements and quantitative indicators [1, 9–11]. The aim of this study was to summarize the major maxillofacial features detected on panoramic radiographs from a relatively large retrospective case series of CCD. Some reliable quantitative indicators that can indicate the presence of disease and help with early diagnosis were presented as the diagnostic reference for dentists.

## Methods

### Patients

This study was approved by the Ethics Committee of West China Hospital of Stomatology, Chengdu, China (WCHSIRB-D-2021-014).

The data records of 30 patients (18 males and 12 females; mean age, 20.53 ± 7.98 years; range, 11–45 years) who were clinically and radiologically diagnosed with CCD were retrospectively collected. Clinical and radiographic data records from December 2013 to December 2020 were obtained from our in-house database. By reviewing health record clinical notes and radiology reports, the patient had a diagnosis of CCD based on (1) Apposed shoulders or deficient clavicles observed on chest radiography. (2) Delayed closure of fontanelles and/or Wormian bone in the cranial suture observed on skull projection. (3) Multiple impacted permanent teeth and/or supernumerary teeth observed on dental radiography. In eight cases, genetic analysis and/or familial hereditary characteristics allowed confirmation of CCD diagnosis. Any patient with orthodontic treatment and maxillofacial surgical treatment that produced morphologic changes of maxillofacial bones, or radiographs presented with inadequate diagnostic quality or incomplete visualization of necessary structures were excluded from enrollment.

For the comparison of quantitative data between CCD patients and healthy individuals, a matched control group was recruited from our picture archiving and communication systems (PACS) and all panoramic radiographs were suitable for measurements. To improve the study reliability, the inclusion criteria of the matched control group were based on the following matching factors: age at the time of panoramic examination, sex, the type of panoramic machine used and exposure parameters. Exclusion criteria for the matched control group were: congenital defect or systemic disease, maxillofacial deformities, orthodontic treatment. Verification of inclusion and exclusion criteria was ensured by reviewing the health records. Because the sample size in the case group was limited (n = 30), we appropriately increased the sample size in the control group (1 case: 10 matched controls), which can minimize the influence of outliers or extreme observations and provide greater statistical power.

All measurements were obtained using the PACS calibration system.

### Panoramic analysis

The case images were captured by various equipment models, including Orthoceph OC200D (Instrumentarium Dental, Tuusula, Finland) at 60 kV, 8.0 mA; PaX-400C (VATECH, Hwaseong, Korea) at 73 kV, 10 mA; and Veraviewepocs (Morita, Kyoto, Japan) at 65 kV, 6 mA and PaX-i (VATECH, Hwaseong, Korea) at 73 kV, 10 mA. It should be noted that the images of the matched controls were captured with the same equipment and parameters as the images of their corresponding case.

#### Dentition states

All panoramic radiographs were recalled to evaluating the states of the dentition. The number and location of the supernumerary teeth and the ratio of impacted or delayed eruption of permanent teeth (except the third molar and supernumerary teeth) were recorded. The impaction and delay of eruption are defined here as the tooth fail to emerge through the top of the alveolar process within normal age range [12].

#### Image measurements

**Fig. 1:**
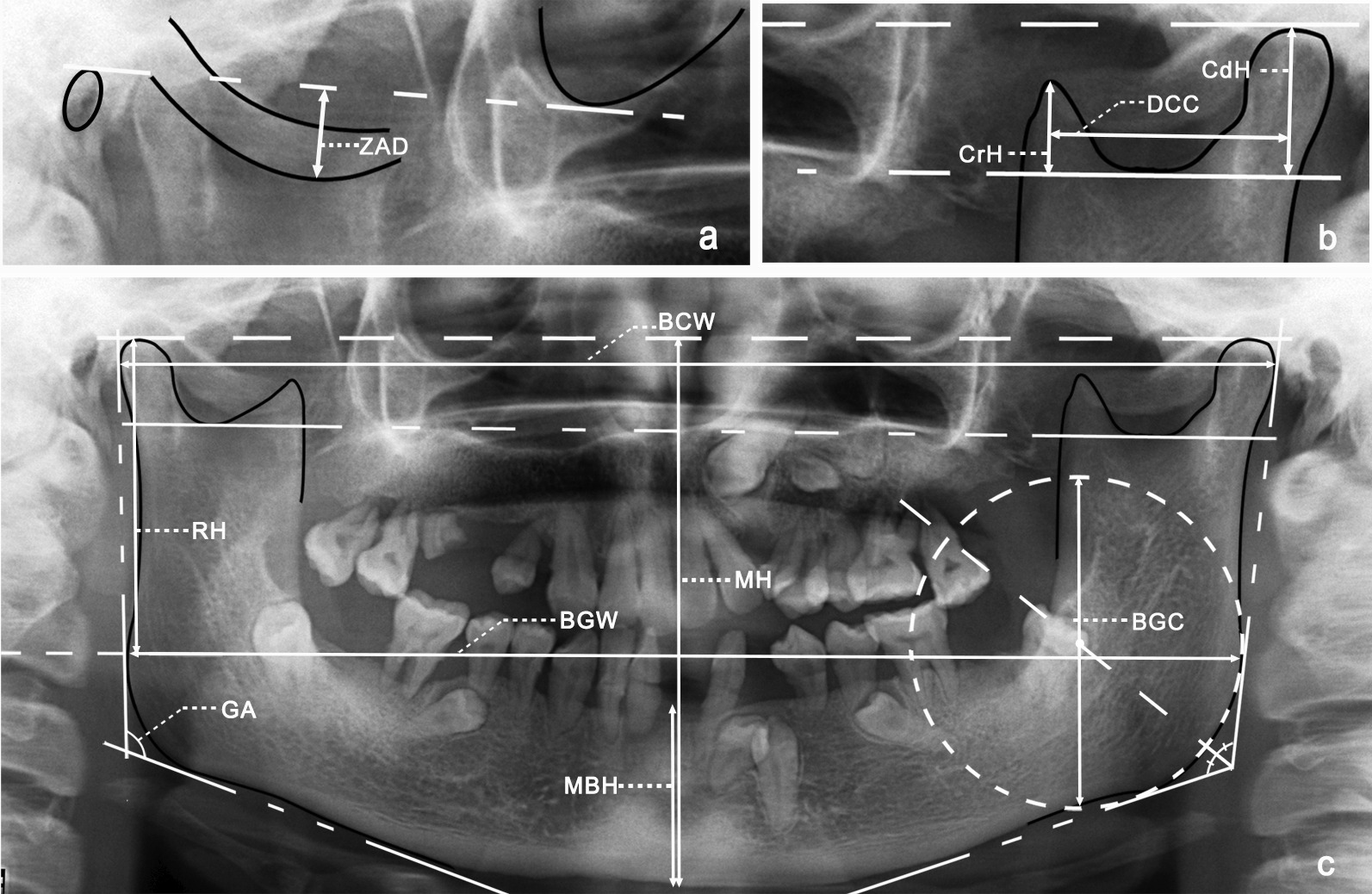
The schematic measuring methods for the panoramic measurements. **a** Cropped zygomatic area indicated the method to measure the ZRD value **b** Cropped upper ramus area indicated the method to measure the CrH, DCC and CdH value **c** Panoramic radiography of a CCD patient indicated the method to measure the BCW, RH, MH, BGW, BGC, GA and MBH value. *ZRD* the degree of the zygomatic arch downward bend; *CrH* coronoid height; *DCC* the distance between coronoid process and condyle; *CdH* condylar height; *BCW* bicondylar width; *RH* ramal height; *MH* mandibular height; *BGW* bigonial width; *BGC* best-fit gonial circle diameter; *GA* gonial angle; *MBH* mandibular body height

A series of panoramic measurements were taken, as described in Table 1 and Fig. 1. The measurements were taken by three investigators. The inter-observer reliability was highly satisfactory, with intraclass correlation coefficient (ICC) greater than 0.82. The CCD group was measured by each investigator. The control group was divided into three parts and each part was measured by an investigator, respectively.

**Table 1:** Measurements in panoramic radiograph evaluation

Measurement	Abbreviation	Description
The degree of the zygomatic arch downward bend	ZAD	Perpendicular distance from the lowest point of the zygomatic arch inferior border to the Frankfort line (drawn from the porion to the orbitale)
Bicondylar width	BCW	Distance between the most external points on the two condyles
Ramal height	RH	Perpendicular distance from the most superior point of the condyle to the line between two gonions
Mandibular height	MH	Perpendicular distance from the gnathion to the line between the most superior points of the bilateral condyles
Mandibular aspect ratio	MAR	Ratio of mandibular height and bicondylar width
Mandibular body height	MBH	Distance from the infradentale to the gnathion
Coronoid height (perpendicular line 1)	CrH	Perpendicular distance from the coronion to the line between the deepest points of the bilateral sigmoid notches
Condylar height (perpendicular line 2)	CdH	Perpendicular distance from the most superior point of the condyle to the line between the deepest points of the bilateral sigmoid notches
The distance between coronoid process and condyle	DCC	Perpendicular distance between perpendicular line 1 and perpendicular line 2
Bigonial width	BGW	Distance between two gonions
Gonial angle	GA	The intersection of line 3 (the tangent to the inferior border of the mandible) and line 4 (the tangent touching the posterior surface of the condyle and ramus)
Best-fit gonial circle diameter	BGC	The best-fit gonial circle:
		Its center located on the Gonial angle bisector
		Tangent to line 3 and line 4
		Fit along the outline of the mandibular arc angle

### Statistical analysis

Measurements of panoramic radiographs were compared between CCD patients and matched controls using the nonparametric Wilcoxon rank-sum test because not all variables were normally distributed (Shapiro–Wilk tests). The P value threshold was set to 0.05. All statistical tests were performed using SPSS software (ver. 19.0 for Windows; SPSS, Inc., Chicago, Illinois).

## Results

### Dentition states

A total of 184 supernumerary teeth were detected in 27 patients (90.0%). The number of supernumerary teeth ranged from 1 to 15, with a mean of 6.8. Regarding the location of these supernumerary teeth, 17 patients (63.0%) presented supernumerary teeth in the incisor region, 27 patients (100.0%) in the premolar region, and 5 patients (18.5%) in the molar region.

**Fig. 2:**
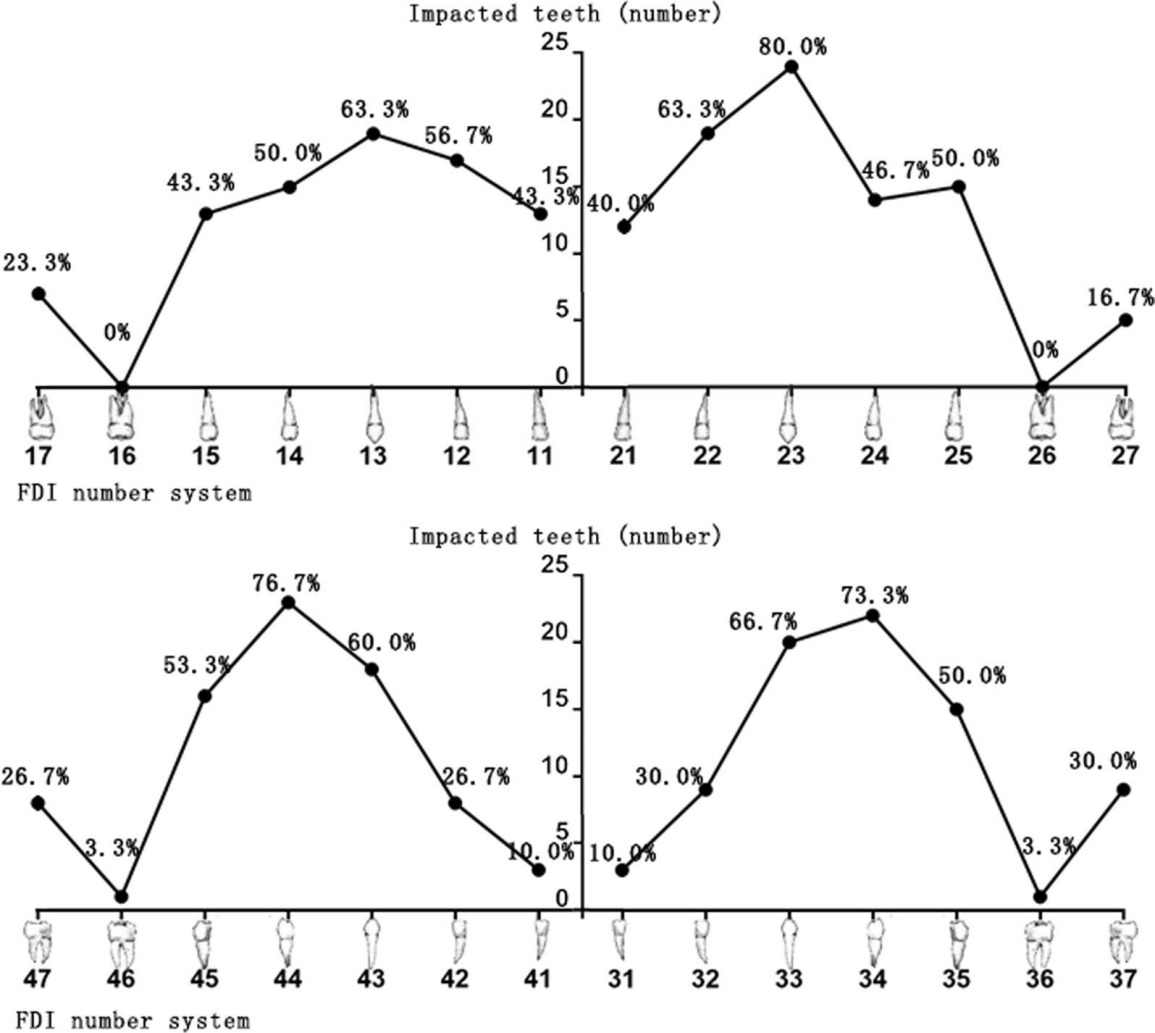
Distribution of the impacted or delayed eruption of permanent teeth in different tooth regions

All patients presented impacted or delayed eruption of some permanent teeth. The number ranged from 2 to 22, with a mean of 11.0. The distribution of these anomalies in different tooth regions is shown in Fig. 2. The lower first premolar (75%) and upper canine (71.7%) were the most frequently impacted teeth. The impaction of the first molar was rare and was present in only one patient.

### Image measurements

The statistical results of measurements in panoramic radiographs are listed in Table 2. Regarding the study design, age and sex were similar between the two groups.

**Table 2:** Comparison of panoramic measurements between the CCD group and the control group

	CCD group (n = 30) (Mean ± SD)	Control group (n = 300) (Mean ± SD)	*P* value
Age, y	20.53 ± 7.98	20.53 ± 7.86	
ZAD, mm			
Left	13.25 ± 3.28	3.62 ± 1.82	< 0.001***
Right	13.21 ± 3.58	3.64 ± 1.87	< 0.001***
BCW, mm	190.19 ± 28.42	192.03 ± 27.96	0.928
RH, mm			
Left	58.84 ± 7.70	56.64 ± 8.92	0.069
Right	57.43 ± 7.58	56.64 ± 9.02	0.357
MH, mm	94.64 ± 13.64	94.49 ± 13.67	0.768
MAR	0.52 ± 0.05	0.51 ± 0.04	0.169
MBH, mm	33.73 ± 5.44	34.33 ± 5.13	0.875
CrH, mm			
Left	11.14 ± 3.71	10.25 ± 2.87	0.077
Right	11.27 ± 3.72	10.25 ± 3.09	0.090
CdH, mm			
Left	18.28 ± 3.42	19.53 ± 4.21	0.176
Right	17.99 ± 3.33	19.27 ± 4.25	0.167
DCC, mm			
Left	22.98 ± 5.55	29.17 ± 5.37	< 0.001***
Right	24.79 ± 5.24	30.10 ± 4.98	< 0.001***
BGW, mm	182.79 ± 27.70	187.01 ± 25.98	0.416
GA, °			
Left	119.37 ± 7.55	119.78 ± 7.11	0.766
Right	118.23 ± 8.34	119.25 ± 6.86	0.337
BGC, mm			
Left	60.32 ± 9.47	37.07 ± 8.04	< 0.001***
Right	60.01 ± 9.04	37.79 ± 8.55	< 0.001***

**Fig. 3:**
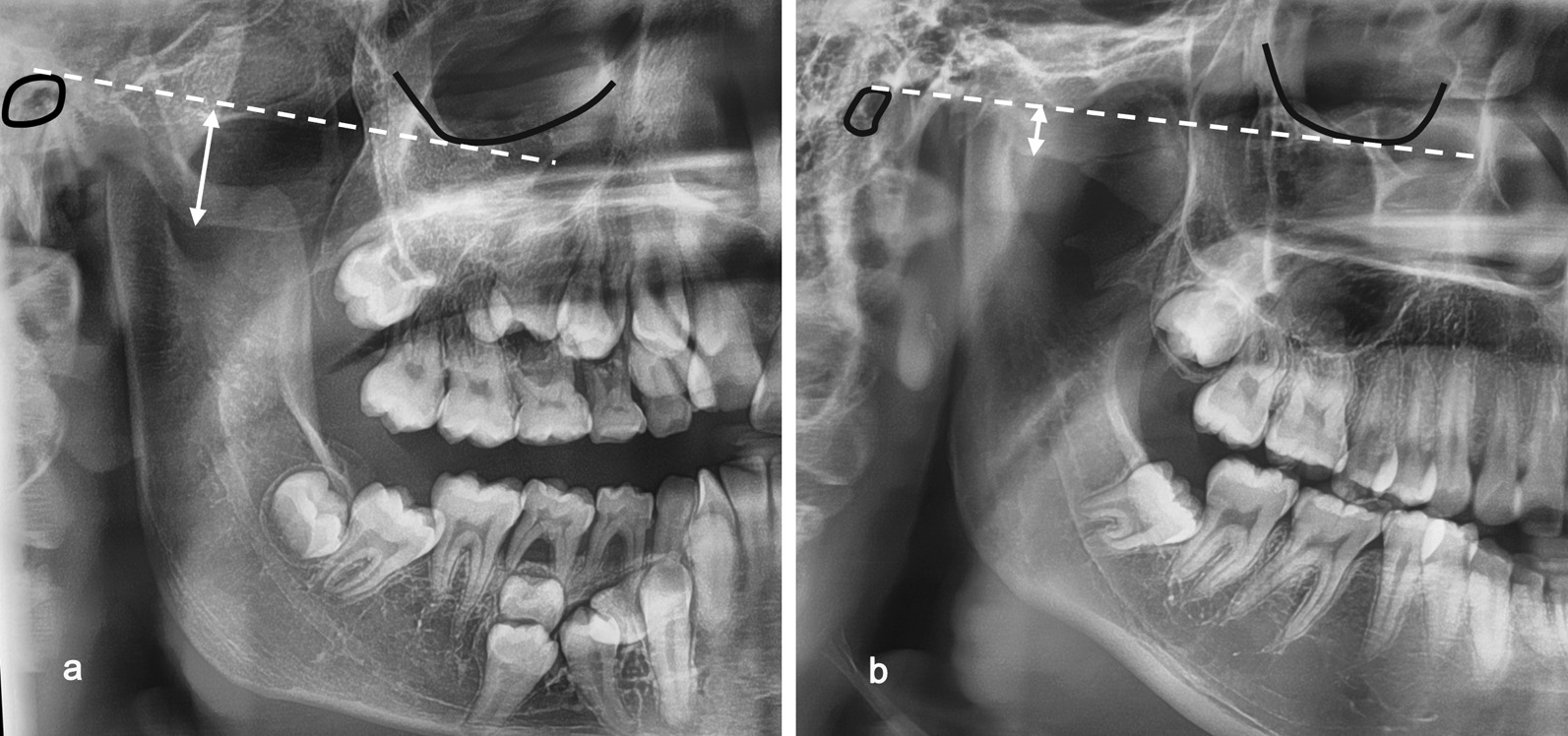
The ZAD observed in the panoramic radiograph. **a** ZAD in a CCD patient **b** normal ZAD in a matched control. The dotted line drawn from the porion to the orbitale indicates the Frankfort line

In our study, all 30 CCD patients with panoramic radiographs showed a downward slope of the zygomatic arch. The degrees of the downward bend between the CCD group and the control group were compared, and both the left and right zygomatic arch were significantly deeper in the CCD group than in the control group (p < 0.001). In the CCD group, the mean values of ZAD on the left and right sides were 13.25 mm and 13.21 mm, respectively; in the control group, these values were 3.62 mm and 3.64 mm. (Fig. 3).

**Fig. 4:**
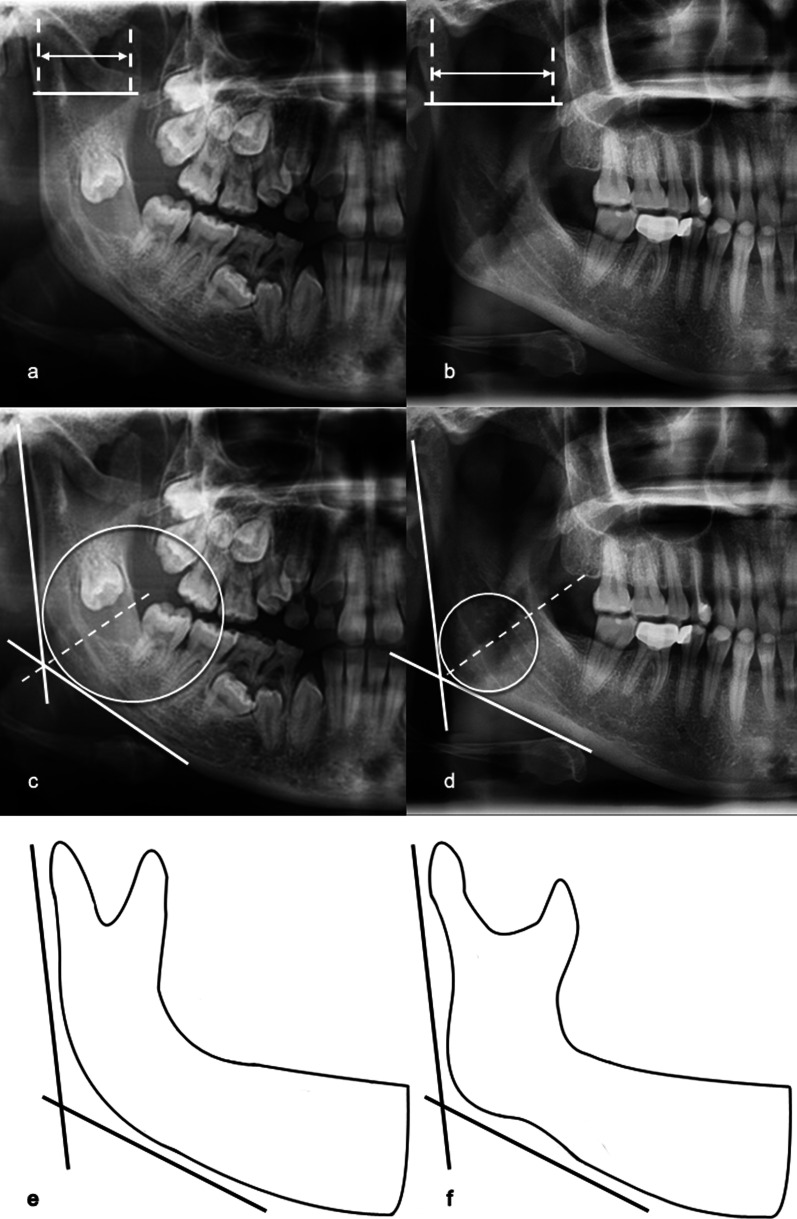
DCC and BGC as observed in a panoramic radiograph. **a** DCC in a CCD patient. **b** Normal DCC in a matched control. **c** BGC in a CCD patient. **d** Normal BGC in a matched control. **e** Schematic representation of the lower curvature of the gonial region in CCD patients. **f** Schematic representation of the normal curvature of the gonial region in matched controls

A comparison of mandibular panoramic measurements revealed that DCC and BGC were significantly different between the two groups. (p < 0.001, Fig. 4). In the CCD group, the mean value of DCC was 22.98 mm on the left side and 24.70 mm on the right side; in the control group, these values were 29.17 mm and 30.10 mm, respectively. The measurement of BGC indicated the difference in the curvature of the mandibular angle region (Fig. 4c–f). The mean values of the left and right sides in the CCD group were 60.32 mm and 60.01 mm, respectively; these values in the control group were 37.07 mm and 37.79 mm, respectively. Other panoramic measurements were not significantly different between the two groups.

**Fig. 5:**
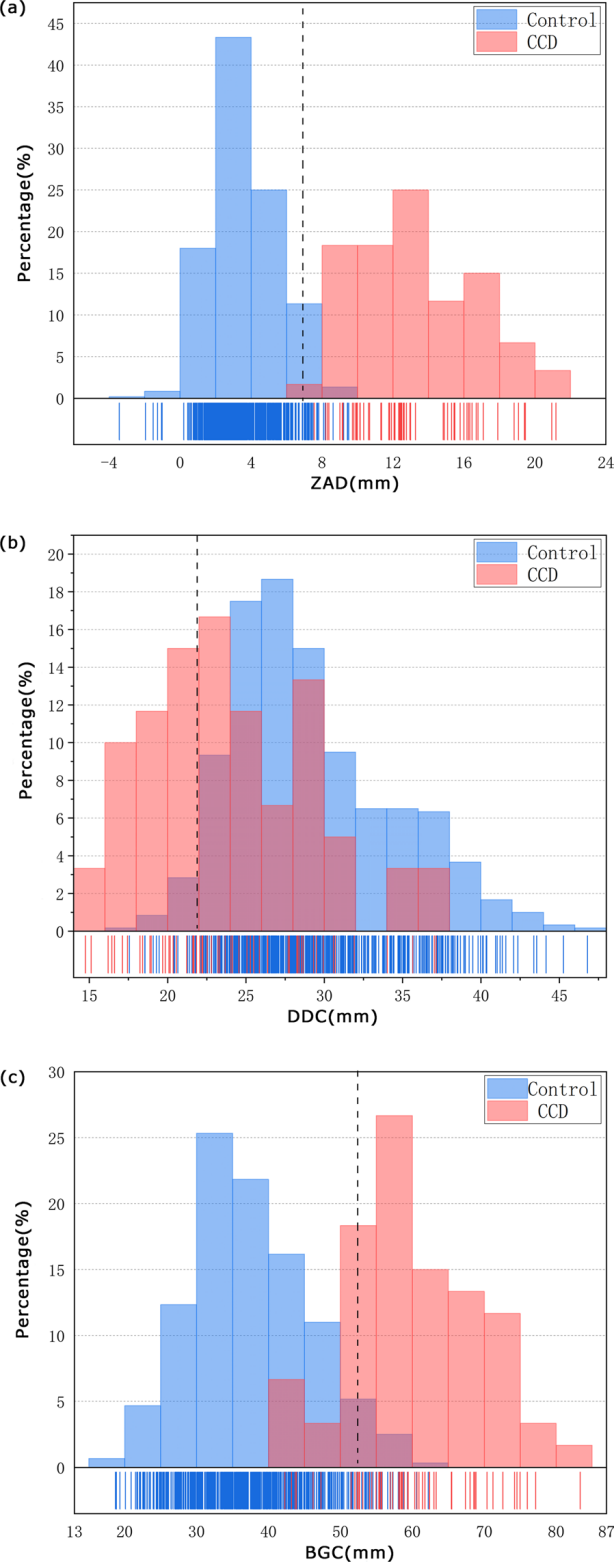
Percentage frequency distribution of 3 panoramic measurement values in the CCD and control groups. **a** Distribution of ZAD values. **b** Distribution of DCC values. **c** Distribution of BGC values. The dotted line indicates the reference cutoff value

Furthermore, Fig. 5a–c depicts the percentage frequency distributions of the ZAD, DCC and BGC values on both the left and right sides via a histogram with a rug plot. The figure indicates that the data range was significantly different between the CCD and control groups. According to the data, the reference cutoff values were established based on the 5th or 95th percentile of the measurements in the control group. The reference cutoff values of ZAD, DCC and BGC were 6.90 mm (upper limit), 22.37 mm (lower limit) and 52.41 mm (upper limit), respectively. In the CCD group, all ZAD values were higher than 6.90 mm, 46.67% of DCC values were less than 22.37 mm, and 83.33% of BGC values were higher than 52.41 mm.

## Discussion

The characteristic manifestations of CCD are skeletal anomalies and irregular dentition [13, 14]. Therefore, some patients who have not yet been diagnosed choose to consult a dental specialist when they perceive their unusual teeth and facial profile. For these patients, dental radiographs can provide valuable diagnostic information. In addition, an early diagnosis will increase opportunities for patients to choose appropriate treatment, to manage complications and to receive genetic counseling [1, 15].

In our study, the presence of supernumerary teeth and impacted teeth were found in 90% and 100% of cases, respectively. Supernumerary teeth and impacted teeth are characteristic feature of CCD patients but exhibits considerable variance, differing in number and position. Meanwhile, the prevalence of non-syndromic supernumerary teeth in Chinese ranges from 1.5 to 10.52%, and 22.15–36.34% patients had multiple supernumerary teeth [16–18]. The prevalence of impacted permanent teeth except the third molar ranges from 6.15 to 8.56% in Chinese [19, 20]. Since these dentition states are not infrequent in the general population, the dentists need more reliable symptoms to improve the diagnostic specificity. The disturbance of intramembranous ossification is regarded as a responsible pathophysiology mechanism of CCD [21]. Therefore, the maxillofacial bone findings are more direct and stable features.

For the first time, this study adopted a series of quantitative assessments in panoramic radiographs to compare the maxillofacial bone features of CCD patients with those of their matched controls. Panoramic radiography enables the visualization of the entire maxillomandibular region on a single film and is commonly used for general dental health evaluation. Panoramic radiography was the most commonly utilized radiographic technique by clinicians when they encountered patients with abnormal dentition [22–25]. There are some inherent problems, such as unequal magnification and image distortion, in panoramic radiographs that can affect the measurements. To attain comparable accuracy, (1) all radiographs included in the study were of excellent diagnostic quality, and (2) each patient had matched controls with the same scanning parameters. Furthermore, the panoramic measurements in the present study, including vertical measurements in the whole area and angular and horizontal measurements in the posterior region, can be obtained with suitable replicability and reliability, which has been revealed by previous studies [26–28].

This study elucidated that the morphology of the zygomatic arch in panoramic radiographs was significantly abnormal in CCD patients. This is in agreement with previous case reports and literature reviews demonstrating that the zygomatic arch may be thin or even discontinuous and has a characteristic downward bend [11, 29–31]. In our study, the Frankfurt line, which has been revealed to have a relatively constant position with the zygomatic arch, was used as the reference line to identify the degree of the zygomatic arch bending downward [32]. The results showed that the ZAD of CCD patients was much larger than that of matched controls, which may be caused by hypoplastic zygomaticotemporal junctions (weak zygomaticotemporal suture) and masseter muscle contraction. The results of measurements in the mandibular angle region further confirm our hypothesis. The masseter muscle originates from the zygomatic arch and extends down to the mandibular angle. Because the patients’ zygomatic arch cannot effectively counter masticatory stress, the vertical component of masseteric force would entail apparent bending of the zygomatic arch. Meanwhile, the masseteric force acting on the mandibular angle region would decrease because of the unstable upper anchorage of the muscle, which can influence the morphology of the mandible in masseteric attachment regions. In our study, the angle measurement (GA) and curvature measurement (BGC) were used to express the morphology of this region. Although there was no statistically significant difference in GA, the CCD patients exhibited a significantly larger BGC with lower curvature, which can reveal morphological anomalies of the mandibular gonial region (Fig. 4e, f). Masseter muscle hypofunction may contribute to this manifestation. The interpretation may be supported by the findings of several soft tissue studies, which revealed a masseter muscle volume reduction in CCD patients [33, 34]. Some clinical case reports revealed their CCD patients present with muscle weakness [35, 36]. Moreover, loss of Runx2 results in reduced expression of pro-myogenic secreted factors, such as Aldh1a2, Igf1, Cxcl12, and Cthrc1, which may affect muscle proliferation and differentiation in mouse model [37]. In our study, it was hypothesized that features on panoramic radiographs were not only determined by bone dysplasia but also influenced by muscle hypofunction. However, the evidence of masticatory muscle electromyographic activity and myopathic changes merits future study.

Another linear measurement (DCC) also showed significant associations with the disease. The results revealed that patients tend to have a narrow ramus. Masticatory muscle function is regarded as one of the determinants of mandibular shape [38]. A previous finding indicated that mandibles with smaller muscle force were characterized by a tall and narrow ramus (more like a parallelogram) [39].

In a clinical setting, when there are several impacted permanent teeth and/or supernumerary teeth on panoramic radiography with diagnostic uncertainty, the contour of the mandible and zygomatic arch should be emphasized. If the characteristic U-shaped mandible and curved zygomatic arch are observed, the ZAD, BGC and DDC values should be measured for comparison with our reference cutoff values.

Regarding the treatment of CCD, surgical-orthodontic traction and orthognathic surgery are the preferred treatments for serious functional and esthetic problems [2, 40, 41]. Nevertheless, previous studies did not consider muscle function as the possible mechanism of osseous changes in CCD. The reconstruction of muscular stability and force balance (e.g., enhancing the strength of the zygomatic arch buttresses) may play an essential role in improving the efficiency of occlusal adjustments and the stability of treatment and esthetic changes. Further study is needed to clarify this possibility.

Our study demonstrated panoramic radiographs had great value in the diagnosis of CCD. However, because of the limitations of 2D images, the limited field of view, magnification, distortion and superposition may adversely affect during clinical implementation. Future work should consider performing more 3D imaging analyses that can comprehensively characterize CCD anomalies.

## Conclusion

Although phenotypic expression can vary from individual to individual, data from the present study demonstrated that CCD patients have some maxillofacial manifestations that can be detected on panoramic radiographs. These features are easy to measure and relatively reliable and therefore may provide valuable information to dentists, particularly in the early diagnosis and timely management of CCD. The possible mechanism of these skeletal features should be considered, as they relate to muscular morphology and strength.

## Data Availability

The datasets generated and/or analyzed during the current study are not publicly available due to the clinical data of all participants in this study belongs to West China Hospital of Stomatology, Sichuan University and we need to obtain the approval of the hospital’s medical department when obtaining it but are available from the corresponding author on reasonable request.

## Abbreviations

CCD: Cleidocranial dysplasia
RUNX2: Runt-related transcription factor 2
PACS: Picture archiving and communication system
